# High Dynamic Range Processing for Magnetic Resonance Imaging

**DOI:** 10.1371/journal.pone.0077883

**Published:** 2013-11-08

**Authors:** Andy H. Hung, Taiyang Liang, Preeti A. Sukerkar, Thomas J. Meade

**Affiliations:** Department of Chemistry, Molecular Biosciences, Neurobiology, Biomedical Engineering, and Radiology, Northwestern University, Evanston, Illinois, United States of America; University of Minnesota, United States of America

## Abstract

**Purpose:**

To minimize feature loss in T_1_- and T_2_-weighted MRI by merging multiple MR images acquired at different T_R_ and T_E_ to generate an image with increased dynamic range.

**Materials and Methods:**

High Dynamic Range (HDR) processing techniques from the field of photography were applied to a series of acquired MR images. Specifically, a method to parameterize the algorithm for MRI data was developed and tested. T_1_- and T_2_-weighted images of a number of contrast agent phantoms and a live mouse were acquired with varying T_R_ and T_E_ parameters. The images were computationally merged to produce HDR-MR images. All acquisitions were performed on a 7.05 T Bruker PharmaScan with a multi-echo spin echo pulse sequence.

**Results:**

HDR-MRI delineated bright and dark features that were either saturated or indistinguishable from background in standard T_1_- and T_2_-weighted MRI. The increased dynamic range preserved intensity gradation over a larger range of T_1_ and T_2_ in phantoms and revealed more anatomical features *in vivo*.

**Conclusions:**

We have developed and tested a method to apply HDR processing to MR images. The increased dynamic range of HDR-MR images as compared to standard T_1_- and T_2_-weighted images minimizes feature loss caused by magnetization recovery or low SNR.

## Introduction

T_1_- and T_2_-weighted imaging are ubiquitous in clinical and research MRI studies [1]. In these imaging methods, contrast can be diminished due to complete magnetization recovery or low signal-to-noise ratio (SNR) [1]–[5]. For MR images with a limited range of T_1_ and T_2_, optimizing the acquisition parameters T_R_ (repetition time) and T_E_ (echo time) circumvents the issue. However, for MR images with a wide range of T_1_ and T_2_ originating from lesions, tissues, or contrast media, feature loss cannot be avoided regardless of the choice of T_R_ and T_E_ [6]. This scenario occurs, for example, when imaging the whole-animal biodistribution of novel contrast agents [7] or labeled biomolecules [8], [9], and can be thought of as a limitation in the “dynamic range” of the imaging method.

**Figure 1:**
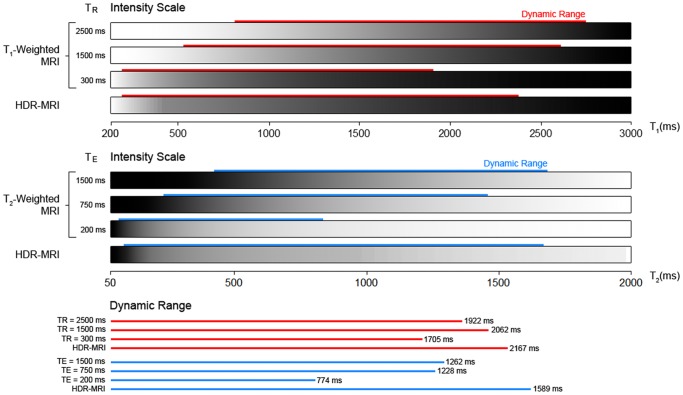
Simulation of intensity scale of T_1_- and T_2_-weighted imaging compared to HDR-MRI. T_1_ and T_2_ values cover the physiological range. For comparative purposes, dynamic range was defined to span from 10% to 90% of each intensity scale. Regions of an image with T_1_ and T_2_ outside this range would appear featureless as either completely white or black. HDR-MRI improved the dynamic range compared to T_1_- and T_2_-weighted imaging. Furthermore, in conventional imaging, dynamic range was sacrificed to visualize short T_1_ and T_2_ features whereas HDR-MRI did not suffer from this limitation.

The formal definition of dynamic range is the ratio between the maximum and the minimum measurable value above noise in an acquisition system [10]. In MRI, T_1_ and T_2_ are not limited by the hardware dynamic range *per se* because they are not directly acquired. However, contrast from a limited range of T_1_ and T_2_ occupies a disproportionately large portion of the signal dynamic range because magnetization recovers and decays nonlinearly according to the two parameters [1]. In other words, voxels with T_1_ and T_2_ values outside of a limited range either vanish into the background noise or saturate the intensity scale (Figure 1) [1]. Therefore, a useful definition of “dynamic range” in T_1_- and T_2_-weighted MRI is the range of T_1_ and T_2_ values that result in detectable and differentiable signal. In an ideal image with unlimited dynamic range as defined here, T_1_ or T_2_ is monotonically represented by intensity. In some other fields of research, dynamic range is defined in a similar way [11], [12].

Dynamic range limitation is a well-recognized issue in the field of photography [10], [13]–[15]. When a scene has a large range of illumination, the resulting photograph invariably has regions that are either saturated or hidden in darkness regardless of the camera exposure time setting. High Dynamic Range (HDR) photography is a technique invented to overcome this challenge by capturing a scene with multiple exposure times and then computationally merging the Low Dynamic Range (LDR) photographs together [16]–[19]. Since the over- or under-exposed regions in any one LDR picture are properly exposed in the other pictures in the set, the merging process produces an HDR image that appears properly exposed throughout. HDR processing is highly automated and has been applied widely to films and photography in the last decade [10], [20]–[25].

We applied HDR processing to merge MR images of different T_R_ and T_E_ combinations in a technique we call High Dynamic Range MRI (HDR-MRI). MR images acquired at different T_R_ and T_E_ are analogous to photographs captured at different exposure times because both produce a set of images with increasing brightness. Similar to HDR photography, HDR-MRI displays more bright and dark features from specimens with a large range of T_1_ or T_2_ compared to standard MRI (Figure 1). This is possible because each T_R_ and T_E_ combination produces an image with complementary information that is then merged to minimize feature loss.

HDR-MRI follows a long history of post-processing algorithms that have been proposed since the 1980s to transform or combine multiple MR images. These algorithms include principle component analysis [26], eigen-images [27], weighted averaging [28], image synthesis [6], [29], and other linear filters [30]–[33]. The processed images aid in diagnostic interpretation by increasing contrast-to-noise ratio (CNR), suppressing interfering features, or allowing dynamic viewing of images synthesized with arbitrary T_R_ and T_E_. HDR-MRI is most similar to image synthesis in that the outputs are readily interpretable by experienced readers, as the hyper- and hypo-intensity relationships of standard acquisitions are preserved. Images produced by other more sophisticated techniques are often more enhanced, but require additional training to read [31]. Compared to image synthesis, HDR-MRI exaggerates or diminishes contrast, is more resistant to uncertainties in T_1_ and T_2_ fitting, and in theory, displays more information simultaneously when shown on an HDR monitor to potentially reduce reading time [34].

To apply HDR processing to MRI, we derived a mathematical expression to transform the MR parameters T_R_ and T_E_ into the HDR parameter exposure value (EV). The performance of HDR-MRI on solution phantoms doped with contrast agents and a live mouse was investigated. Compared to standard MRI, HDR-MRI differentiated signals from a larger range of contrast agent concentrations in phantoms and revealed more anatomical features *in vivo*.

## Materials and Methods

### Ethics Statement

Animal studies were conducted in accordance with the National Institutes of Health Guide for the Care and Use of Laboratory Animals. The protocol was approved by the Northwestern Institutional Animal Care and Use Committee (Permit Number: 2010–2027).

### Theory

A limitation in optical image capture technology is the low dynamic range of films and sensors relative to the human eye [14], [15]. As a result, some features are obscured in darkness while others appear saturated. HDR processing overcomes this limitation by merging multiple LDR photos with varying exposure times to capture the full range of features observed naturally by the human eye. There are a number of algorithms for HDR processing [16], [17], [35]–[37]. The commercial package utilized here (HDR Pro built into Adobe Photoshop CS5) implements the algorithm developed by Debevec and Malik [16].

**Figure 2:**
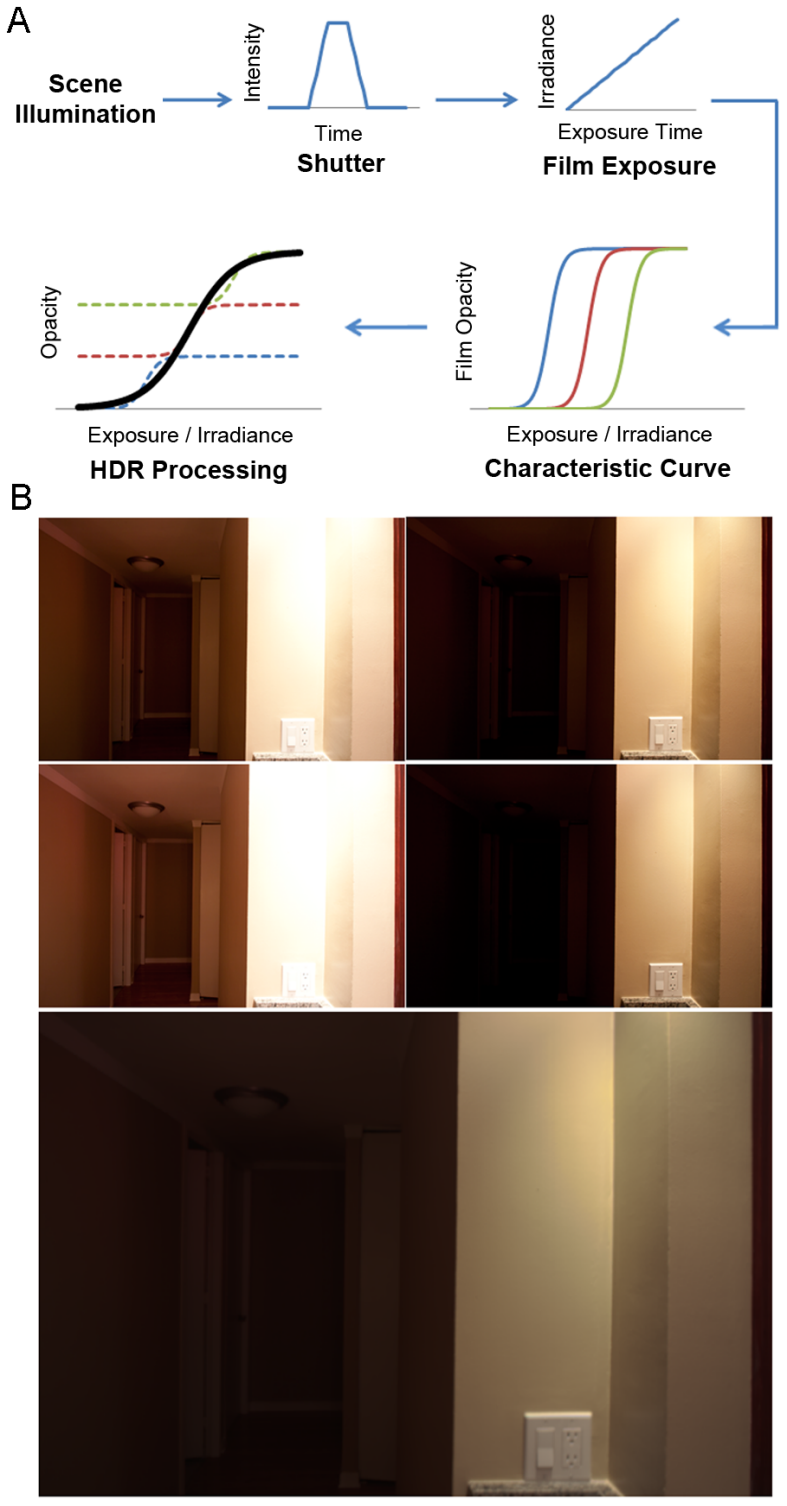
HDR processing in photography. (A) LDR photos taken at varying exposure times produce shifted characteristic curves (otherwise known as the digital conversion function *f*) that cover different ranges of irradiance. HDR processing merges the LDR characteristic curves to produce an HDR characteristic curve that covers a larger range of irradiance. The HDR characteristic curve is used to calculate object illumination to alleviate the issues of over- and under-exposure associated with conventional LDR photography. (B) An example showing the merger of 4 LDR photos (smaller photos) to a HDR photo (larger photo). In the HDR photo, the front wall showed no saturation from over-exposure while the poorly lit hallway remained visible. This was not achieved in any single LDR photo due to the limited dynamic range.

The Debevec algorithm estimates the physical illumination (I) of an object based on the principles of digital image capture systems. An image is captured through light sensor exposures (E) that scale with object illumination and exposure time (t) (Equation 1).
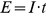
(1)The light sensor converts the exposure (E) to a finite range of pixel brightness through a digital conversion function *f*, otherwise known as the characteristic curve (Figure 2A) (Equation 2).
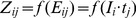
(2)Z_ij_ denotes the brightness of pixel i at exposure time t_j_. With the reasonable assumption of *f* as a monotonically increasing function, an inverse function, *f* ^−1^, exists (Equation 3).
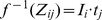
(3)Taking the natural logarithm defines the function g (Equation 4)
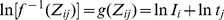
(4)In Equation 4, g and I_i_ are the unknowns and can be estimated by minimizing a quadratic error function (Equation 5).
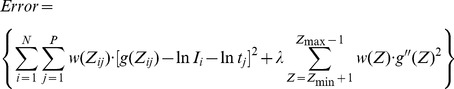
(5)N is the number of pixels per picture and P is the number of pictures to be merged. When N and P are sufficiently large, the error function can be seen as an over-determined system of equations that is solved readily using available algorithms [16]. The first term of the error function ensures fidelity of Equation 4. The second term ensures smoothness of g. The strength of the second term is controlled by the choice of λ. The second derivative g″(Z) is defined in discrete form (Equation 6).
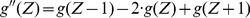
(6)The weighing function, w, emphasizes pixels closer to the center of the light sensor dynamic range so that pixels without signal or that are saturated weigh less in the determination of g (Equations 7 and 8).
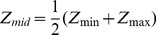
(7)

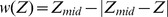
(8)Once g has been computed, the estimation of physical illumination I_i_ can be further improved by a weighted average (Equation 9).
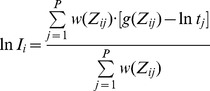
(9)In this equation, the determination of I_i_ is weighted toward pixels with proper exposure because the weighing function approaches zero when the pixel is under- or over-exposed (i.e. Z_ij_ approaches Z_min_ or Z_max_). The re-constructed HDR image presents the calculated physical illumination (I) of the scene and consequently spans over a wider dynamic range than any of the source images (Figure 2B).

To utilize HDR processing for MR images, it is necessary to transform the MRI parameters T_R_ and T_E_ into the photography parameter exposure time (t). T_R_ and T_E_ in MRI are analogous to exposure time in photography because they are acquisition parameters that affect voxel intensity just as exposure time affects pixel intensity. For a Multi-Echo Spin Echo (MESE) pulse sequence, the voxel signal intensity is governed by Equation 10 [1], [38].
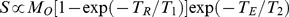
(10)M_o_ is the equilibrium magnetization, T_R_ is the repetition time, T_E_ is the echo time, and T_1_ and T_2_ are the longitudinal and transverse relaxation time, respectively. In this equation, T_R_ and T_E_ have monotonic relationships with the MR signal (S), analogous to the relationship between exposure time (t) and film exposure (E) in photography (Equation 1) [39].

By setting equations 1 and 10 equal to each other, Equation 11 is obtained:
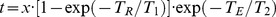
(11)x is a constant consisting of M_o_ and I. The calculated t can be used to solve Equation 5, with the index for t_j_ running from 1 to 4 to represent images acquired at 4 different T_R_s or T_E_s used in the experiments. In practice, t is further transformed into exposure values (EV) by Equation 12 for input into the HDR software [16], [39].
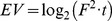
(12)F refers to the relative aperture. Since EV is a relative value in HDR processing, F and, to a first order approximation of constant M_o_, x can be arbitrarily chosen. We set F to 4 because it is a commonly used setting in photography and x to 1 for simplicity. It is important to note that the T_1_, T_2_, and M_o_ chosen to calculate t affect processing. This complication should not significantly impact the qualitative interpretation of HDR-MR images because it does not alter the hyper- and hypo-intensity relationships in the image. The effect of non-uniform T_1_, T_2_, and M_o_ on HDR processing is examined more closely in the discussion section.

### High Dynamic Range Processing

For T_1_-weighted image series with varying T_R_, the RecoSeries macro in ParaVision software (version 5.1, Bruker BioSpin, Billerica, MA) was used to reconstruct all 4 images on the same intensity scale. This pre-processing was not required for images in T_E_ series because they were acquired in a single scan. All images were converted from signed 16-bit integer encoding to unsigned 16-bit integer encoding in ImageJ (NIH, Bethesda, MD).

T_1_ and T_2_ of solution phantoms were fitted using the built-in Image Sequence Analysis tool in ParaVision or a custom program written in Matlab (ver. R2010b, MathWorks, Natick, MA). HDR processing was performed using HDR Pro built into Adobe Photoshop CS5. The only input parameters for processing were EV and the choice of white point. The EV of each source image was calculated using Equations 11 and 12 based on T_1_ and T_2_ values specified in Table S1. The T_1_ and T_2_ used represent intermediate values within the estimated range for each figure. The white point of the intensity scale was always set to the brightest pixel of the image. No additional adjustments, such as tone mapping or detail enhancement, were performed. The output was saved in 32-bit TIFF format. For the phantom results, each HDR image was normalized using ImageJ such that the intensity of the water signal is equivalent to the water signal of the brightest input image. All images were displayed on the same intensity scale within each figure except for the in vivo images. For pseudo-coloring, Fire LUT in ImageJ was applied.

### Simulation of Dynamic Range of T_1_-weighted, T_2_-weighted, and High Dynamic Range MRI

The T_1_- and T_2_-weighted intensity scales were created by simulation in Matlab using equation 10. T_E_ = 0 and T_R_ = ∞ was used for T_1_- and T_2_-weighted simulation, respectively. Each scale is displayed between pixel values 0 and 65535. The HDR intensity scale was generated by merging the LDR scales using HDR processing as described previously.

### Gd(III)HPN_3_DO3A and Iron Oxide Nanoflower Phantom MRI

The MR contrast agents used in these studies were synthesized as previously described [40], [41]. Solution phantoms were prepared by placing 25 µL of Gd(III)HPN_3_DO3A or iron oxide Nanoflower (NF) samples in flame-sealed 0.6 mm ID 9″ glass Pasteur pipettes. The stock concentrations of Gd(III)HPN_3_DO3A and NF were 0.5 M and 4.8 mg/mL, respectively. Serial dilutions of 10 fold were performed 6 times for each agent. All images were acquired using a 7.05 T Bruker PharmaScan fitted with a RF RES 300 1H 089/023 quadrature transceiver volume coil (Bruker BioSpin, Billerica, MA). The MESE pulse sequence was used in all acquisitions. For T_1_-weighted HDR, the imaging parameters were T_R_ = 200, 400, 800, 1600 ms, T_E_ = 10.635 ms, NEX = 1, Bandwidth = 195 Hz/pixel, FOV = 23×23 mm^2^, slice thickness = 1 mm, and matrix size = 256×256. For T_2_-weighted HDR, the imaging parameters were T_R_ = 4000 ms, T_E_ = 10.635…531.75 ms in 10.635 ms increments, NEX = 1, Bandwidth = 391 Hz/pixel, FOV = 23×23 mm^2^, slice thickness = 1 mm, and matrix size = 128×128.

### Quantification

Image intensity quantification was done using ImageJ with the Measure function on ROIs. Normalized contrast was defined by Equation 13.
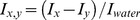
(13)I_x,y_ is the normalized contrast between samples x and y. It is calculated by subtracting the intensity of sample y (I_y_) from that of sample x (I_x_), followed by normalization with the intensity of water (I_water_). To calculate SNR, water signal was divided by noise either in the void region or within the water signal as indicated in the figures. Noise in the void is calculated as 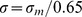, where *σ* is the physical noise and *σ_m_* is the measured standard deviation, to account for the Rician distribution of noise in MR magnitude images [42]. Noise within water is approximated as *σ = σ_m_* and requires no correction because the noise distribution is largely Gaussian at high SNR. Error bars represent *σ* that were propagated from the source images according to the arithmetic operations performed. For example, the *σ* of I_x_ - I_y_ is calculated as 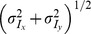.

### *In vivo* Imaging

A female Balb/C athymic nude mouse was acquired from Harlan (Indianapolis, IN) and housed under pathogen-free conditions. The mouse was maintained under anesthesia (1–3% isoflurane) and respiration monitor. Tubing containing heated water was positioned under the mouse to maintain constant body temperature. Imaging was performed on an 89 mm bore PharmaScan 7.05 T MR imager fitted with shielded gradient coils (Bruker BioSpin, Billerica, MA, USA) using a RF RES 300 1H 089/038 quadrature transceiver volume coil (Bruker BioSpin, Billerica, MA, USA). Respiration-gated MESE scans with fat suppression were used: T_R_ = 5632 ms, T_E_ = 10.635…212.7 ms in 10.635 ms increments, NEX = 1, Bandwidth = 391 Hz/pixel, FOV = 35×35 mm^2^, slice thickness = 1 mm, and matrix size = 128×128. HDR processing was performed as described previously. The T_2_ map was generated by fitting every voxel in the T_E_ series to A·exp(−T_E_/T_2_)+C using Matlab. Masking was done by manually thresholding the T_E_ 21 LDR image at 1500 such that the voxels below and above the threshold were set to 0 and 1, respectively. The intensity scale of each image was adjusted individually for visibility.

## Results

**Figure 3:**
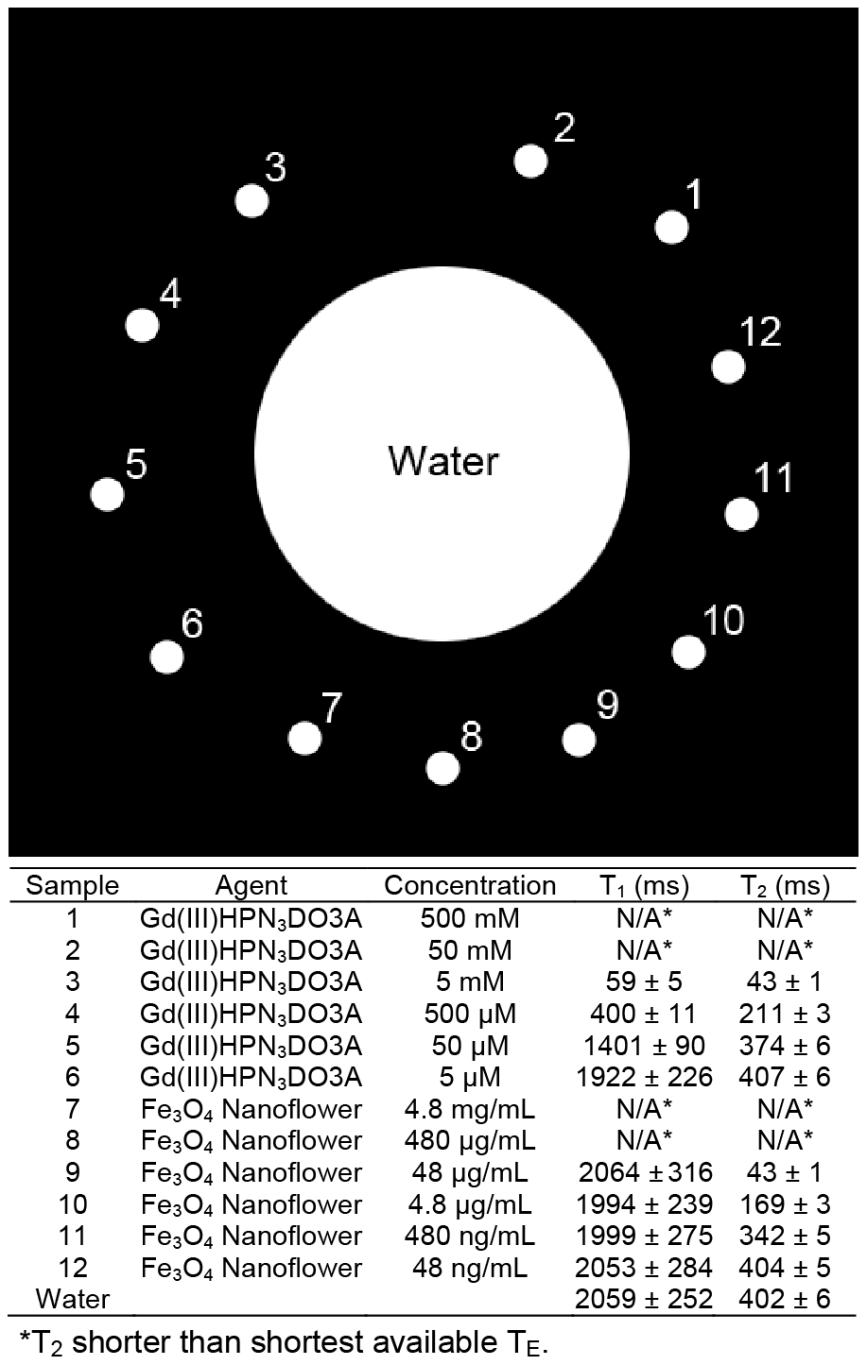
Legends and characterizations of solution phantom samples 1–12.

To assess the applicability of HDR processing to MR images, we prepared solution phantoms consisting of T_1_ (Gd(III)HPN_3_DO3A) [41] and T_2_ (NF) [40] contrast agents (Figure 3). This assortment of phantoms simulated scenarios when many different tissues or a large range of contrast agent concentrations are imaged in the same view. The concentrations, T_1_, and T_2_ values of each phantom are listed in Figure 3. For samples with high concentrations of contrast agents (samples 1, 2, 7, and 8), the T_2_ was short compared to the shortest T_E_ available for the pulse sequence used. As a result, these samples were invisible in most of the MR images acquired [1], [38].

### HDR Processing of MR Images

**Figure 4:**
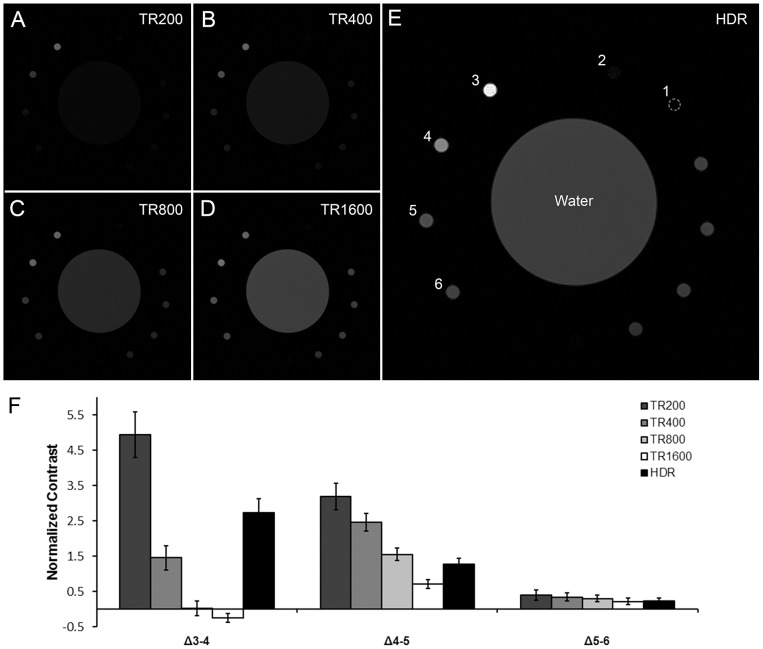
T_1_-weighted HDR-MRI. A series of images were acquired with constant T_E_ at 10.635 ms and varying T_R_ at (A) 200 ms (B) 400 ms (C) 800 ms and (D) 1600 ms. Images A–D were computationally merged by the HDR algorithm to generate the (E) HDR-MR image. The HDR image accentuated the contrast between samples 3 and 4 without suppressing samples 5 and 6 into the background. None of the LDR images, A–D, captured both features simultaneously. While image A had the largest normalized contrast, it also had the poorest SNR. Conversely, images with better SNR had worse normalized contrast, most notably between samples 3 and 4. HDR-MRI combined the complementary features of each image. (F) Quantification of contrast normalized against water. Error bars represent standard deviation.

HDR-MR images were generated and compared to conventional images. For T_1_-weighted HDR-MRI, images were taken at constant T_E_ and varying T_R_. The result showed that a T_R_ of 200 ms gave the highest contrast among samples 3–5, while longer T_R_s better distinguished the lower concentration samples from background (Figure 4A–D). By merging the LDR images, HDR processing combined the advantage of pronounced contrast at short T_R_ and improved delineation of weak signals at longer T_R_ (Figure 4E, F).

**Figure 5:**
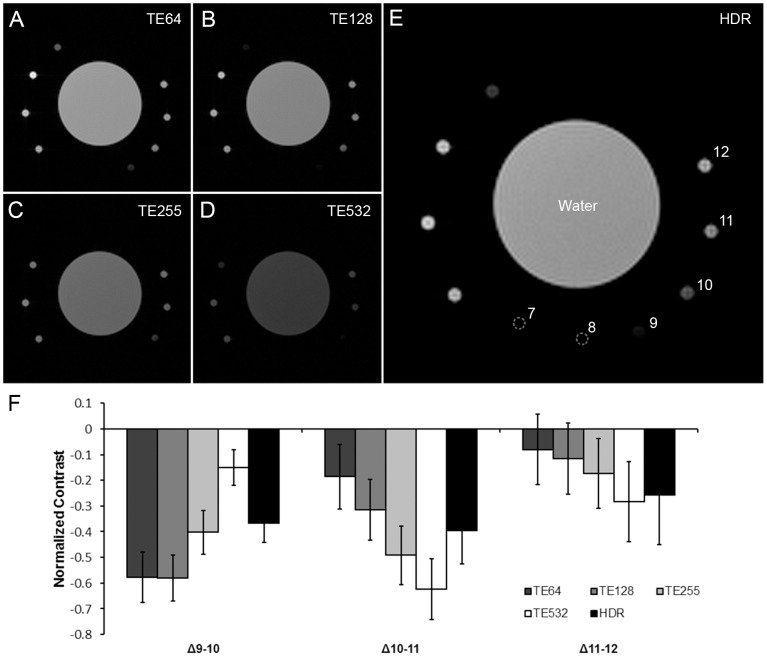
T_2_-weighted HDR-MRI. A series of images were acquired with constant T_R_ at 4000 ms and T_E_ at (A) 63.81 ms (B) 127.62 ms (C) 255.24 ms and (D) 531.75 ms. Images A–D were computationally merged by the HDR algorithm to generate the (E) HDR image. HDR-MRI accurately captured the intensity difference between samples 9, 10, 11, and 12, which was not achieved in any of the source images. This was shown quantitatively by contrasts normalized against water (F). Image A was poor at differentiating samples 10–12, B and C were poor at differentiating samples 11 and 12, and D was poor at differentiating samples 9 and 10. Error bars represent standard deviation.

Similarly, for T_2_-weighted HDR-MRI using images with varying T_E_, each image spanned a different dynamic range and provided the optimal contrast between different pairs of samples (Figure 5A–D). For example, the T_E_ used in Figure 5A and 5D are best at differentiating sample 9 from 10 and sample 10 from 11, respectively. The HDR image had an extended dynamic range and was capable of differentiating samples 9, 10, and 11, on the same image (Figure 5E, F). However, this wider dynamic range sacrificed quantitative contrast between samples since the absolute range of pixel intensities is fixed by the display device and file encoding capability.

### EV Interval Selection

The EV interval of the source LDR images has a significant impact on the quality of the final HDR image. In the extreme, a selection of LDR images with identical EV produces a HDR image without any enhancement. In photography, the optimal EV interval is typically between 1 and 2 to ensure overlap of the characteristic curves at different exposure times (Equation 2, Figure 2A) and a wide dynamic range [16], [43]. To investigate whether MR images have similar optimal EV gaps, a series of images with constant T_R_ and varying T_E_ were acquired; selections of 4 single LDR images with EV intervals of 0, 0.5, 1, and 1.4 were utilized for HDR processing. Each selection had a different T_E_ range that centered around T_E_ = 270 ms, with the largest spanning from 11 ms to 521 ms (Table S1). In addition, simple averaging of 4 LDR images with T_E_ 11 ms and 521 ms were performed to compare with HDR processing.

**Figure 6:**
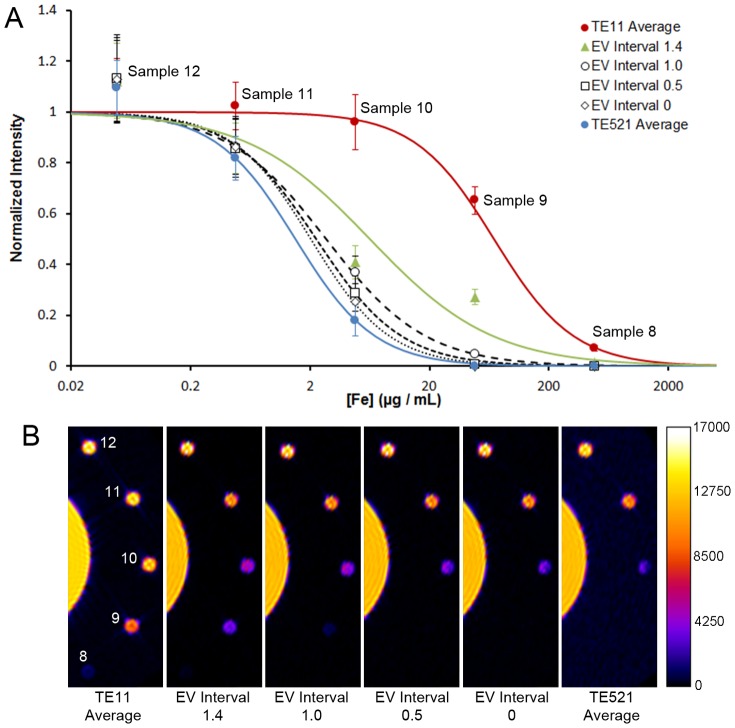
EV interval analysis. (A) Signal intensities normalized against water at various NF concentrations. Sigmoidal curves illustrate trends in the data and can be thought of as characteristic curves. As the EV interval increased, the characteristic curve slope flattened to span over a wider dynamic range. The characteristic curves of the two averaged LDR images (T_E_11 and T_E_521) had the steepest slopes and were analogous to the EV interval 0 condition. (B) Comparison between averaged LDR images and HDR images. The HDR image with EV interval 1.4 showed contrast between every sample from 8 to 12, indicating its large dynamic range. N = 4 was used for the averaged images. Error bars represent standard deviation.

As previously discussed, HDR improves the dynamic range of an image to include dark and bright features by the sacrifice of relative contrast, and this is further illustrated in Figure 6A. The curves shown can be interpreted as rough representations of characteristic curves (pixel intensity vs. film exposure) because iron concentration is correlated to T_2_. The two LDR curves had similar slopes, but were shifted horizontally relative to each other, indicating similar dynamic ranges that cover different regions. With HDR processing using EV interval 0, the slope was similar to the LDR images, consistent with expectation; as the EV interval increased, the curve flattened to span through a wider range of concentrations, indicating an increase in dynamic range with a concomitant decrease in relative contrast. The same conclusion regarding EV intervals could be drawn visually from the acquired images (Figure 6B). Wider EV intervals generated images with greater dynamic range that better delineated low signal features from background while retaining differential contrast in the high signal features. It is evident that an EV gap of 1–2 yielded the best combination of range and contrast, similar to the guidelines used in photography. Functionally, the increased dynamic range of HDR-MRI as compared to conventional imaging allowed for the capture of contrast enhancement from a larger range of contrast agent concentrations.

### *In Vivo* Imaging and HDR

**Figure 7:**
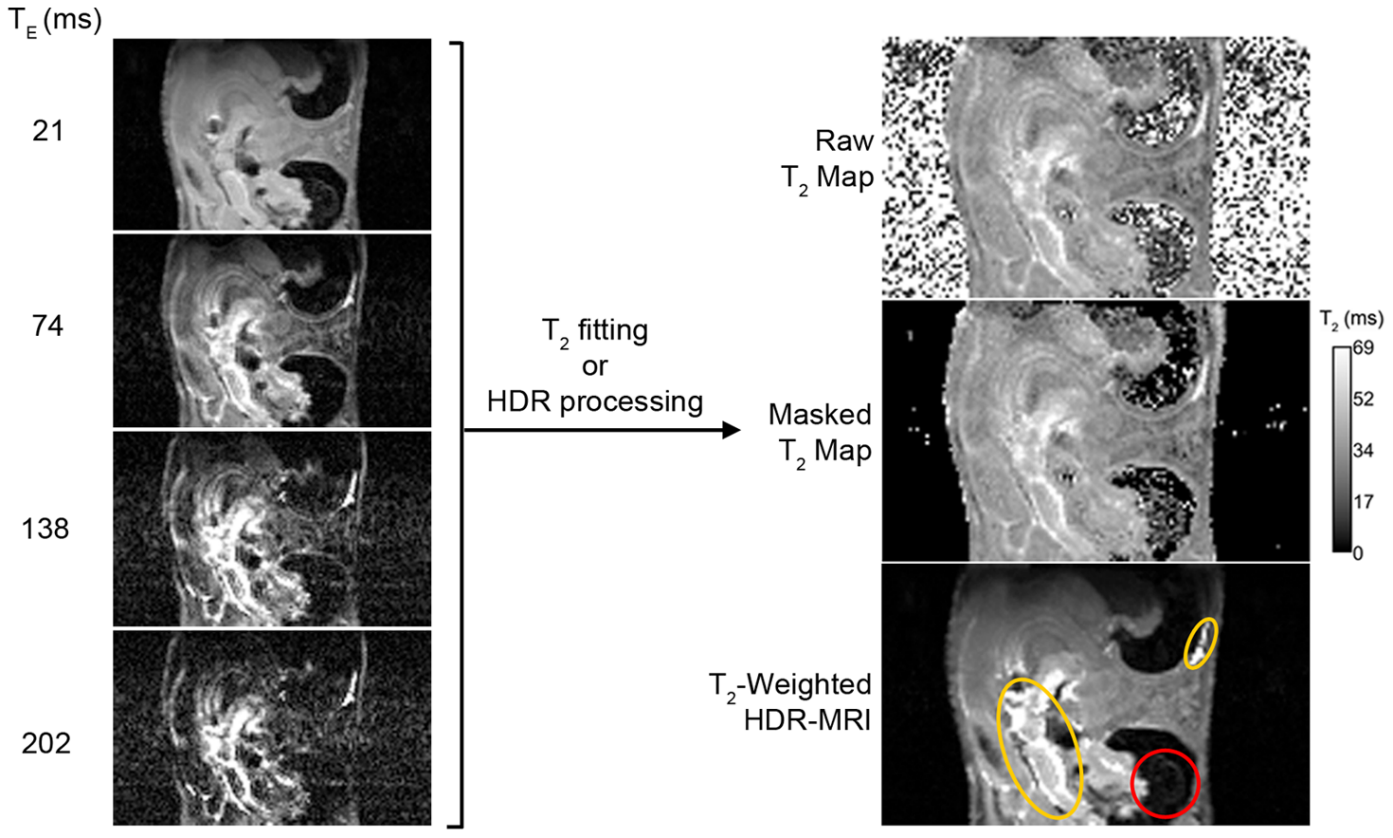
*In vivo* HDR-MRI. A series of images were acquired with constant T_R_ at 5632 ms and varying T_E_ as indicated. The same four LDR images were used to generate both the T_2_ map and the HDR-MR image. Masking of the T_2_ map was done by manual thresholding. In the HDR image, red and yellow outlines highlight features that were not captured in one or more of the individual LDR images. HDR-MRI captures the same features as T_2_ mapping, but is less noisy in the low signal regions. Low signal features can be accurately depicted in HDR-MRI even when the features are only visible in a single LDR image.

Based on the *in vitro* findings, a live mouse was scanned using a carefully selected T_E_ series that was merged into a T_2_-weighted HDR image (Figure 7). The HDR image contains features that one or more of the individual LDR images lack. For example, the red highlight shows the low signal features present when T_E_ = 21 ms but not at longer T_E_s, and the yellow highlight outlines the contrast between features that were only evident when T_E_≥74 ms. For comparison, a T_2_ map was generated using the same dataset. The T_2_ map generally captures the same features, but required manual masking and is noisier in the regions with low SNR due to insufficient signal for fitting at long T_E_. A similar result was obtained when T_2_ was mapped using images acquired at T_E_ = 21, 43, 64, and 85 ms to improve accuracy (Figure S1). In contrast, HDR processing is able to capture low signal features accurately even in the case when they are only visible in a single source image.

## Discussion

Our results demonstrate a method of applying HDR processing to MRI. HDR-MRI exchanges relative contrast for dynamic range to minimize feature loss caused by complete magnetization recovery and low SNR. The improved dynamic range captured contrast agent enhancements over a larger concentration range and preserved more anatomical details *in vivo*. These properties make HDR-MRI suitable for imaging a large number of tissue types and contrast agent concentrations simultaneously, such as when performing small animal biodistribution studies by MRI.

### The Effect of T_1_, T_2_, and M_o_ on HDR Processing

When EV is calculated using Equations 11 and 12, the analogy between photography and MR imaging is complicated by the fact that each voxel has a different T_1_, T_2_, and M_o_. One way to understand the effect of this complication is to think of the inputted EV as under- and over-estimating the EV of different voxels. Consequently, their physical illuminations become miscalculated according to Equations 4 and 9. In the case when T_1,voxel_<T_1,input_, T_2,voxel_>T_2,input_, or M_o,voxel_>M_o,median_, the HDR algorithm overestimates the illumination of the voxel (Figure S2). Incidentally, shorter T_1_, longer T_2_, and larger M_o_ all lead to higher signals on an MR image to coincide with the overestimated illumination (Equation 10). Similarly, when T_1,voxel_>T_1,input_, T_2,voxel_<T_2,input_, or M_o,voxel_<M_o,median_, the HDR algorithm underestimates the voxel illumination. In other words, the effect of voxel-dependent T_1_, T_2_, and M_o_ can be understood as a brightening of high signal features and a darkening of low signal features. The result is the exaggeration and diminishment of quantitative contrast without alteration to the hyper- and hypo-intensity relationships between the different features.

To validate the theoretical expectations and quantify the contrast modifications, HDR processing was performed on the same set of source images using EVs calculated from different T_1_ and T_2_ combinations (Figures S3 and S4). Only the choice of T_1_ affected the processing of T_1_-weighted HDR-MRI because T_E_≪T_2_ and was fixed across the T_R_ series (Equation 11 and Figure S3). Similarly, only the choice of T_2_ was important in T_2_-weighted HDR-MRI (Figure S4). The result showed that in both cases, the quantitative contrast varied, but the relative feature brightness remained the same regardless of the EV used, consistent with the theoretical predications.

A further assessment showed that the degree of intensity variation depended on the choice of T_1_ or T_2_ in calculating the input EV. When extreme values of T_1_ or T_2_ were used, the contrast modification can be significant. For example, when T_2_ = 43 ms was used to calculate EV in the T_2_-weighted HDR-MR image, the water illumination (T_2_ = 400 ms) was overestimated to such a degree that the darker features became invisible in comparison (Figure S4). However, when intermediate T_1_ or T_2_ was chosen, the variations were mitigated. For example, in the T_2_-weighted HDR-MR image (T_2_ range = 43–400 ms), EVs calculated using a T_2_ anywhere between 170–370 ms resulted in intensity variations of no more than 17% relative to water (Figure S4). This degree of variation is commonly experienced in conventional T_2_-weighted imaging as a result of operator differences in choosing T_E_ (Figure S4). A similar conclusion can be drawn for T_1_-weighted HDR-MRI (Figure S3).

In HDR-MRI, voxel-dependent T_1_, T_2_, and M_o_ results in the exaggeration or diminishment of quantitative contrast while conserving hyper- and hypo-intensity relationships in accordance with the source images. When intermediate T_1_ or T_2_ is used to calculate the input EV, the contrast modifications introduced are no greater than the variations introduced by operator differences in conventional imaging due to T_R_ and T_E_ choices. Similar outputs can be obtained using a range of T_1_ or T_2_ in the calculation of EV, making the method somewhat robust. Based on these observations, we expect the effect of voxel-dependent EV to only minimally interfere with qualitative interpretation, especially on viewing software with dynamic brightness and contrast adjustments. If quantitative information is desired, a more sophisticated approach could be taken in the future to modify the HDR algorithm based on the MR signal equation.

### The Effect of T_R_ and T_E_ Choice on HDR Processing

In HDR photography, the choice of exposure times affects the resulting HDR image. Similarly, the choice of T_R_s or T_E_s is an important consideration in HDR-MRI. The choice of T_R_s in T_1_-weighted HDR-MRI and T_E_s in T_2_-weighted HDR-MRI determines the EVs and, in turn, the dynamic range of the output (Figure 6). For an MR image set that covers a fixed dynamic range, variations in T_R_ or T_E_ change the calculated EV for each image (Equation 11); as a result, the feature intensities in the HDR-MR image would vary in a manner similar to when the choice of T_1_ or T_2_ is varied in the calculation of EV. As discussed previously, this degree of variation is not expected to hinder qualitative interpretation. To obtain unique HDR-MR images for quantitative purposes, the underlying HDR algorithm would need to be modified.

### Recommendations

**Figure 8:**
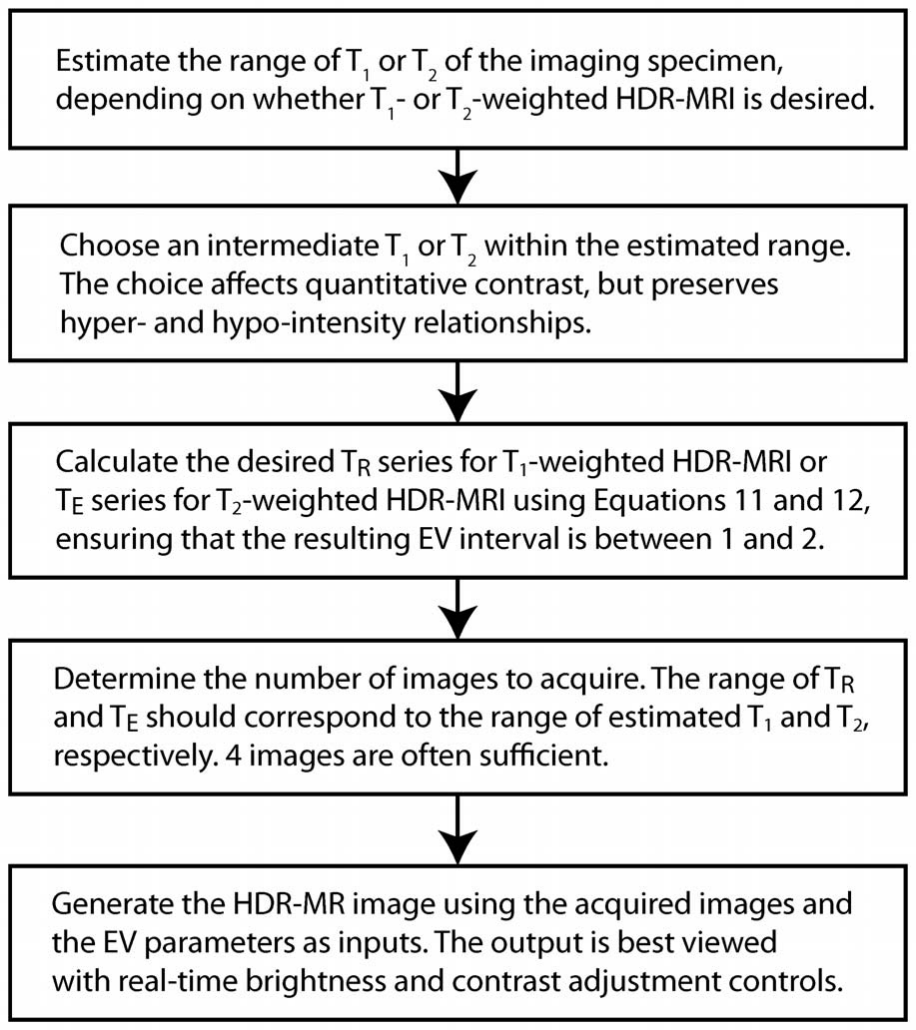
Recommended protocol for HDR-MRI when utilizing a commercial package.

Based on the findings presented, a recommended protocol for HDR-MRI is summarized in a flowchart (Figure 8). Choosing an intermediate T_1_ or T_2_ value for EV calculation and a T_R_/T_E_ series with EV intervals between 1–2 have been shown to work well with the commercial package utilized here. Although no precise method is prescribed for choosing T_1_ or T_2_ in calculating EV and some operator differences cannot be avoided, similar results are obtained within a range. Consequently, the choice should not significantly impact qualitative interpretation based on hyper- and hypo-intensity relationships. Once the EV interval has been determined, the number of images to acquire depends on the estimated range of T_1_ or T_2_ in the image. For best results, the shortest and the longest T_R_ or T_E_ in the sequence should approximate the shortest and the longest estimated T_1_ or T_2_, respectively. In our experience, four images comfortably cover many scenarios.

### Cost Benefit Analysis

HDR-MRI preserves features with extreme T_1_ or T_2_ values at the expense of relative contrast and imaging time compared to standard T_1_- and T_2_-weighted imaging. Relative contrast in HDR-MRI is decreased due to flattening of the characteristic curve such that an expanded portion of the intensity scale is used to represent T_1_ and T_2_ contrast. With the advent of HDR displays, this limitation of HDR-MRI may be alleviated. HDR displays exhibit a contrast ratio of 50000∶1 and a maximum intensity of 8500 cd/m^2^, compared to a typical desktop display with a contrast ratio of 300∶1 and a maximum intensity of 300 cd/m^2^ [34]. Trading contrast for feature visibility using HDR-MRI would be advantageous on an HDR display due to the ample availability of contrast.

Compared to single image acquisitions, T_1_-weighted HDR-MRI lengthens imaging time by 7 to 8 fold while T_2_-weighted HDR-MRI has no additional cost. The reason is that T_1_-weighted HDR-MRI uses long T_R_s while T_2_-weighted HDR-MRI uses a multi-echo sequence that acquires in a single T_R_. Therefore, the time cost of HDR-MRI is the same as in T_1_ and T_2_ mapping.

For the same amount of imaging time or with the same set of source images, many algorithms can be applied to generate synthetic images of higher quality. A simple way to improve quality is by averaging multiple images to obtain increased SNR and CNR (contrast-to-noise ratio). A direct comparison between averaging and HDR-MRI in terms of SNR is difficult because of the complex SNR behavior produced by the HDR algorithm (Figure S5). However, HDR-MRI likely depicts features at the extremes of the T_1_/T_2_ scale with higher contrast due to the use of different T_R_ and T_E_ combinations.

Compared to T_1_ and T_2_ mapping, HDR-MRI is more resistant to noise but does not provide quantitative parameters. Uncertainty in the fitting of voxel T_1_ or T_2_ does not affect HDR processing because the individual voxel relaxation times are not used as parameters. In addition, Equation 4 guarantees that the low SNR voxels that are problematic for relaxation time fitting would always remain dark in the HDR-MR image.

Transformations based on component analysis are another method of combining MR images [26], [27], [30], [31], [33]. These algorithms enhance the CNR of the features of interest and can additionally suppress the intensities of other interfering features. HDR-MRI does not achieve the CNR improvement of these methods. Instead, HDR-MRI produces outputs similar to T_1_- and T_2_-weighted images that are familiar to experienced readers and is suitable when the appearance of the feature of interest is not known *a priori*. In this regard, HDR-MRI is closely related to image synthesis [6], [29]. Image synthesis is a method to dynamically generate synthetic T_1_ or T_2_-weighted images at arbitrary T_R_ and T_E_ using a map of relaxation times. In theory, HDR-MRI compresses the information of all possible synthetic images into one image at the expense of relative contrast. On an HDR display, the higher information content of HDR-MR images may reduce reading time without sacrificing accuracy.

HDR processing can be easily built into viewing software to provide an alternative way of visualizing multi-image data alongside other algorithms. If the images have already been acquired for a different purpose such as relaxation time mapping, the cost of HDR-MRI is purely computational. In addition to the use of HDR processing with the MESE pulse sequence used here, it is possible to apply the same principle to other contrast parameters or pulse sequences such as flip angle, inversion time, phase-based flow imaging, or diffusion-weighted imaging.

## Conclusions

HDR processing is a powerful technique in photography used to simultaneously capture dark and bright features. In MRI, having a large range of T_1_ or T_2_ results in diminished feature visibility, analogous to the challenge faced in photography. We have applied the fundamental principles of HDR photography to MR imaging and provided both phantom and *in vivo* examples showing the comparisons between standard T_1_- or T_2_-weighted images and HDR-MR images. HDR-MRI provides an alternative to standard imaging by merging multiple non-optimal images highlighting different features into a single image that displays all features simultaneously. This technique may increase contrast agent visibility in whole-animal biodistribution studies and reduce diagnostic workload by maximizing the information content of an image.

## Supporting Information

Figure S1 **Additional comparison between T_2_ mapping and T_2_-weighted HDR-MRI.** T_2_ mapping and HDR-MRI based on the same source images display similar features, with the T_2_ map appearing noisier in the low signal regions. A similar result was obtained when a more accurate T_2_ map was produced using shorter T_E_s.
(TIF)

Figure S2 **Qualitative picture for the effect of T_1_, T_2_, and M_o_ on HDR Processing.** In the presented scenario, there are only two voxels. The inputted exposure times t_j_, j = 1–5 (or more strictly, EVs) are accurate for voxel A. Voxel B is physically brighter. (A–C) The g function *before* HDR processing when illumination (I) is arbitrarily assumed to be 1 by the algorithm at both voxel A and B. (A) In photography, voxels A and B share the same set of five exposure times. Therefore, the inputted t_j_ are accurate for both A and B. Voxel B is physically brighter, resulting in its larger voxel intensities. I_B_ can be accurately estimated by the algorithm. (B) If the input overestimates the true exposure times of voxel B, as when T_1,voxel_>T_1,input_, T_2,voxel_<T_2,input_, or M_o,voxel_<M_o,median_ (Equation 11), I_B_ is underestimated (Equations 4 and 9). (C) Conversely, if the input underestimates the true exposure times of voxel B, as when T_1,voxel_<T_1,input_, T_2,voxel_>T_2,input_, or M_o,voxel_>M_o,median_, I_B_ is overestimated. (D) In all three cases, HDR processing calculates I_B_ to obtain the identical final monotonically increasing g function (Equations 4 and 5).
(TIF)

Figure S3 **Effects of the choice of T_1_ and T_2_ in calculating EV on T_1_-weighted HDR processing.** The values tested are the T_1_ and T_2_ of the samples imaged. The choice of T_2_ has almost no effect while the choice of T_1_ has moderate effect. In general, the quantitative contrast changes, but the relative order of the feature intensities is preserved regardless of the T_1_ chosen for EV calculation. The variations caused by the different choices of T_1_ are similar to variations seen in conventional T_1_-weighted imaging due to different T_R_ settings. Water signal has been normalized to 100 across all images.
(TIF)

Figure S4 **Effects of the choice of T_1_ and T_2_ in calculating EV on T_2_-weighted HDR processing.** The values tested are the T_1_ and T_2_ of the samples imaged. The choice of T_1_ has almost no effect while the choice of T_2_ has moderate effect. In general, the conclusion is the same as for T_1_-weighted HDR processing. The quantitative contrast changes, but the relative order of the feature intensities is preserved regardless of the T_2_ chosen for EV calculation. The variations caused by the different choices of T_2_ are similar to variations seen in conventional T_2_-weighted imaging due to different T_E_ settings. Water signal has been normalized to 100 across all images.
(TIF)

Figure S5 **SNR comparison between image averaging and HDR-MRI.** Data from Figure 6 was used for the analysis. Dashed line (----) and dotted line (····) represent the SNR of the averaged LDR images with T_E_ 11 ms and T_E_ 521 ms, respectively. (A) When calculated against void noise, HDR-MRI SNR improved with increasing EV interval and outperformed averaging. (B) When calculated against noise in the water signal, HDR-MRI SNR remained constant across EV intervals and underperformed averaging. SNR is inhomogeneous in HDR-MRI because the characteristic curve is nonlinear and each voxel is processed by the algorithm independently. N = 4 was used for the averaged images. Error bars represent standard deviation.
(TIF)

Table S1 **T_1_ and T_2_ used in the calculation of EV parameters for HDR processing.**
(DOCX)

## References

[1] Mansson S, Bjornerud A (2001) Physical principles of medical imaging by nuclear magnetic resonance. In: Merbach AE, Toth E, editors. The chemistry of contrast agents in medical magnetic resonance imaging. Chichester: John Wiley and Sons, Ltd. pp. 1–43.

[2] Harney AS, Meade TJ (2010) Molecular imaging of in vivo gene expression. Future Med Chem 2: 503–519. 21426178 10.4155/fmc.09.168 PMC4507573

[3] Hu FQ, Joshi HM, Dravid VP, Meade TJ (2010) High-performance nanostructured MR contrast probes. Nanoscale 2: 1884–1891. 20694208 10.1039/c0nr00173b PMC3110061

[4] Modo M, Beech JS, Meade TJ, Williams SCR, Price J (2009) A chronic 1 year assessment of MRI contrast agent-labelled neural stem cell transplants in stroke. Neuroimage 47: T133–T142. 18634886 10.1016/j.neuroimage.2008.06.017 PMC4145694

[5] Lin WB, Hyeon T, Lanza GM, Zhang MQ, Meade TJ (2009) Magnetic nanoparticles for early detection of cancer by magnetic resonance imaging. MRS Bull 34: 441–448. 26166945 10.1557/mrs2009.120 PMC4495966

[6] Andreisek G, White LM, Theodoropoulos JS, Naraghi A, Young N, et al (2010) Synthetic-echo time postprocessing technique for generating images with variable T2-weighted contrast: Diagnosis of meniscal and cartilage abnormalities of the knee. Radiology 254: 188–199. 20032152 10.1148/radiol.2541090314

[7] Ye Z, Wu XM, Tan MQ, Jesberger J, Grisworld M, et al (2013) Synthesis and evaluation of a polydisulfide with gddota monoamide side chains as a biodegradable macromolecular contrast agent for MR blood pool imaging. Contrast Media Mol Imaging 8: 220–228. 23606425 10.1002/cmmi.1520 PMC4060968

[8] Sriram R, Lagerstedt JO, Petrlova J, Samardzic H, Kreutzer U, et al (2011) Imaging apolipoprotein ai in vivo. NMR Biomed 24: 916–924. 21264979 10.1002/nbm.1650 PMC3726305

[9] Frias JC, Lipinski MJ, Lipinski SE, Albelda MT (2007) Modified lipoproteins as contrastagents for imaging of atherosclerosis. Contrast Media Mol Imaging 2: 16–23. 17318917 10.1002/cmmi.124

[10] Reinhard E, Heidrich W, Debevec P, Pattanaik S, Ward G, et al.. (2010) High dynamic range imaging: Acquisition, display, and image-based lighting. 2nd ed. Burlington, MA: Morgan Kaufmann. pp. 1–18.

[11] Vo-Dinh T, Cullum B (2000) Biosensors and biochips: Advances in biological and medical diagnostics. Fresenius J Anal Chem 366: 540–551. 11225766 10.1007/s002160051549

[12] Wang ZD, Lee JH, Lu Y (2008) Label-free colorimetric detection of lead ions with a nanomolar detection limit and tunable dynamic range by using gold nanoparticles and dnazyme. Adv Mater 20: 3263–3267.

[13] Pattanaik SN, Tumblin J, Yee H, Greenberg DP (2000) Time-dependent visual adaptation for fast realistic image display. SIGGRAPH; 2000; New Orleans, LA. ACM. pp. 47–54.

[14] Ferwerda JA, Pattanaik SN, Shirley P, Greenberg DP (1996) A model of visual adaptation for realistic image synthesis. SIGGRAPH; 1996; New Orleans, LA. ACM. pp. 249–258.

[15] Dowling JE (1987) The retina: An approachable part of the brain. 1st ed. Cambridge, MA: Belknap Press of Harvard University Press. pp. 187.

[16] Debevec PE, Malik J (1997) Recovering high dynamic range radiance maps from photographs. SIGGRAPH; 1997; Los Angeles, CA. ACM. pp. 369–378.

[17] Mann S, Picard RW (1995) On being ‘undigital’ with digital cameras: Extending dynamic range by combining differently exposed pictures. IS&T annual conference; 1995; Washington, DC. Society for Imaging Science and Technology. pp. 422–428.

[18] Grossberg MD, Nayar SK (2003) Determining the camera response from images: What is knowable? IEEE Trans Pattern Anal Mach Intell 25: 1455–1467.

[19] Grossberg MD, Nayar SK (2004) Modeling the space of camera response functions. IEEE Trans Pattern Anal Mach Intell 26: 1272–1282. 15641715 10.1109/TPAMI.2004.88

[20] Debevec P (1998) Rendering synthetic objects into real scenes: Bridging traditional and image-based graphics with global illumination and high dynamic range photography. SIGGRAPH; 1998; Orlando, FL. ACM. pp. 189–198.

[21] Debevec P, Hawkins T, Tchou C, Duiker HP, Sarokin W, et al.. (2000) Acquiring the reflectance field of a human face. SIGGRAPH; 2000; New Orleans, LA. ACM. pp. 145–156.

[22] Imai FH (2009) Reviewing state-of-art imaging modalities and its potential for biomedical applications. J Dent 37: e7–e14. 19523739 10.1016/j.jdent.2009.05.006

[23] Jacobs K, Loscos C, Ward G (2008) Automatic high-dynamic range image generation for dynamic scenes. IEEE Comput Graph Appl 28: 84–93. 10.1109/mcg.2008.23 18350936

[24] Ward G (2003) Fast, robust image registration for compositing high dynamic range photographs from hand-held exposures. Journal of Graphics Tools 8: 17–30.

[25] Petschnigg G, Szeliski R, Agrawala M, Cohen M, Hoppe H, et al (2004) Digital photography with flash and no-flash image pairs. ACM Transaction on Graphics 23: 664–672.

[26] Schmiedl U, Ortendahl DA, Mark AS, Berry I, Kaufman L (1987) The utility of principal component analysis for the image display of brain-lesions - a preliminary, comparative-study. Magn Reson Med 4: 471–486. 3600253 10.1002/mrm.1910040508

[27] Windham JP, Abdallah MA, Reimann DA, Froelich JW, Haggar AM (1988) Eigenimage filtering in MR imaging. J Comput Assist Tomogr 12: 1–9. 3335646 10.1097/00004728-198801000-00001

[28] Lee JN, Riederer SJ (1987) The contrast-to-noise in relaxation-time, synthetic, and weighted-sum MR images. Magn Reson Med 5: 13–22. 3657492 10.1002/mrm.1910050103

[29] Riederer SJ, Suddarth SA, Bobman SA, Lee JN, Wang HZ, et al (1984) Automated MR image synthesis - feasibility studies. Radiology 153: 203–206. 6089265 10.1148/radiology.153.1.6089265

[30] Brown DG, Lee JN, Blinder RA, Wang HZ, Riederer SJ, et al (1990) CNR enhancement in the presence of multiple interfering processes using linear filters. Magn Reson Med 14: 79–96. 2191179 10.1002/mrm.1910140109

[31] Soltanian-Zadeh H, Windham JP (1992) Novel and general approach to linear filter design for contrast-to-noise ratio enhancement of magnetic resonance images with multiple interfering features in the scene. J Electron Imaging 1: 171–182.

[32] Buxton RB, Greensite F (1991) Target-point combination of MR images. Magn Reson Med 18: 102–115. 2062223 10.1002/mrm.1910180112

[33] Zhu XP, Hutchinson CE, Hawnaur JM, Cootes TF, Taylor CJ, et al (1994) Magnetic-resonance image synthesis using a flexible model. Br J Radiol 67: 976–982. 8000842 10.1259/0007-1285-67-802-976

[34] Seetzen H, Heidrich W, Stuerzlinger W, Ward G, Whitehead L, et al (2004) High dynamic range display systems. ACM Trans Graph 23: 760–768.

[35] Robertson MA, Borman S, Stevenson RL (2003) Estimation-theoretic approach to dynamic range enhancement using multiple exposures. J Electron Imaging 12: 219–228.

[36] Robertson MA, Borman S, Stevenson RL (1999) Dynamic range improvement through multiple exposures. Proceedings of the 1999 International Conference on Image Processing; 1999. IEEE. pp. 159–163.

[37] Mitsunaga T, Nayar SK (1999) Radiometric self calibration. IEEE Conference on Computer Vision and Pattern Recognition; 1999. IEEE. pp. 374–380.

[38] Caravan P, Ellison JJ, McMurry TJ, Lauffer RB (1999) Gadolinium(III) chelates as MRI contrast agents: Structure, dynamics, and applications. Chem Rev 99: 2293–2352. 11749483 10.1021/cr980440x

[39] Jacobson RE, Ray SF, Attridge CG, Axford NR (2000) The manual of photography: Photographic and digital imaging. Woburn, MA: Focal Press.

[40] Hu FQ, MacRenaris KW, Waters EA, Schultz-Sikma EA, Eckermann AL, et al (2010) Highly dispersible, superparamagnetic magnetite nanoflowers for magnetic resonance imaging. Chem Commun 46: 73–75. 10.1039/b916562b PMC4125028 20024297

[41] Mastarone DJ, Harrison VSR, Eckermann AL, Parigi G, Luchinat C, et al (2011) A modular system for the synthesis of multiplexed magnetic resonance probes. J Am Chem Soc 133: 5329–5337. 21413801 10.1021/ja1099616 PMC3086647

[42] Gudbjartsson H, Patz S (1995) The rician distribution of noisy MRI data. Magn Reson Med 34: 910–914. 8598820 10.1002/mrm.1910340618 PMC2254141

[43] Kao WC (2008) High dynamic range imaging by fusing multiple raw images and tone reproduction. IEEE Trans Consum Electron 54: 10–15.

