# Supplementary figures and images for: High Dynamic Range Processing for Magnetic Resonance Imaging
Source: PLoS One. 2013 Nov 8;8(11):e77883. doi: 10.1371/journal.pone.0077883 (PMC3826760; doi:10.1371/journal.pone.0077883)

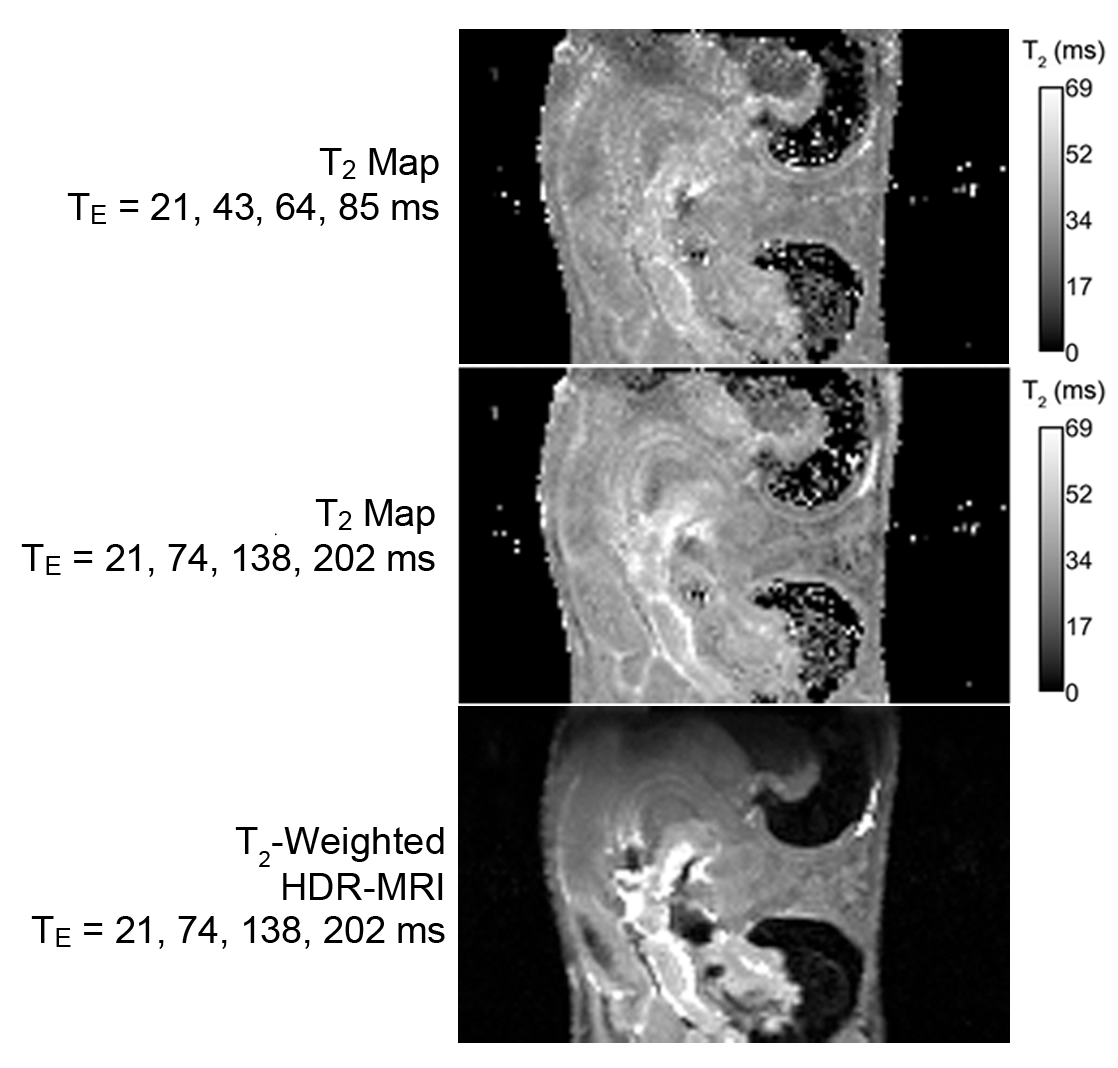

Supplement: Figure S1 — Additional comparison between T2 mapping and T2-weighted HDR-MRI. T2 mapping and HDR-MRI based on the same source images display similar features, with the T2 map appearing noisier in the low signal regions. A similar result was obtained when a more accurate T2 map was produced using shorter TEs. (TIF) [file pone.0077883.s001.tif]

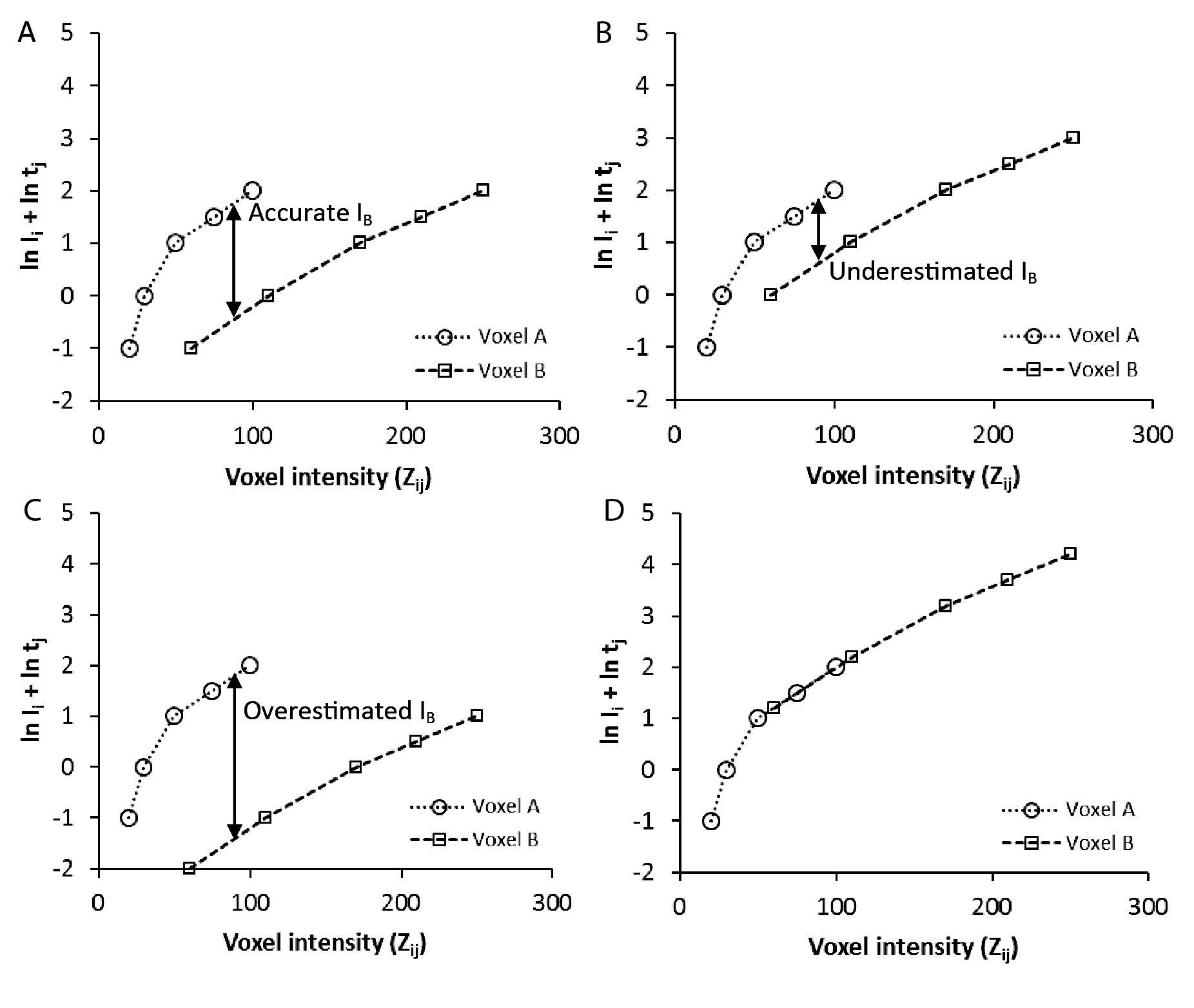

Supplement: Figure S2 — Qualitative picture for the effect of T1, T2, and Mo on HDR Processing. In the presented scenario, there are only two voxels. The inputted exposure times tj, j = 1–5 (or more strictly, EVs) are accurate for voxel A. Voxel B is physically brighter. (A–C) The g function before HDR processing when illumination (I) is arbitrarily assumed to be 1 by the algorithm at both voxel A and B. (A) In photography, voxels A and B share the same set of five exposure times. Therefore, the inputted tj are accurate for both A and B. Voxel B is physically brighter, resulting in its larger voxel intensities. IB can be accurately estimated by the algorithm. (B) If the input overestimates the true exposure times of voxel B, as when T1,voxel>T1,input, T2,voxel<T2,input, or Mo,voxel<Mo,median (Equation 11), IB is underestimated (Equations 4 and 9). (C) Conversely, if the input underestimates the true exposure times of voxel B, as when T1,voxel<T1,input, T2,voxel>T2,input, or Mo,voxel>Mo,median, IB is overestimated. (D) In all three cases, HDR processing calculates IB to obtain the identical final monotonically increasing g function (Equations 4 and 5). (TIF) [file pone.0077883.s002.tif]

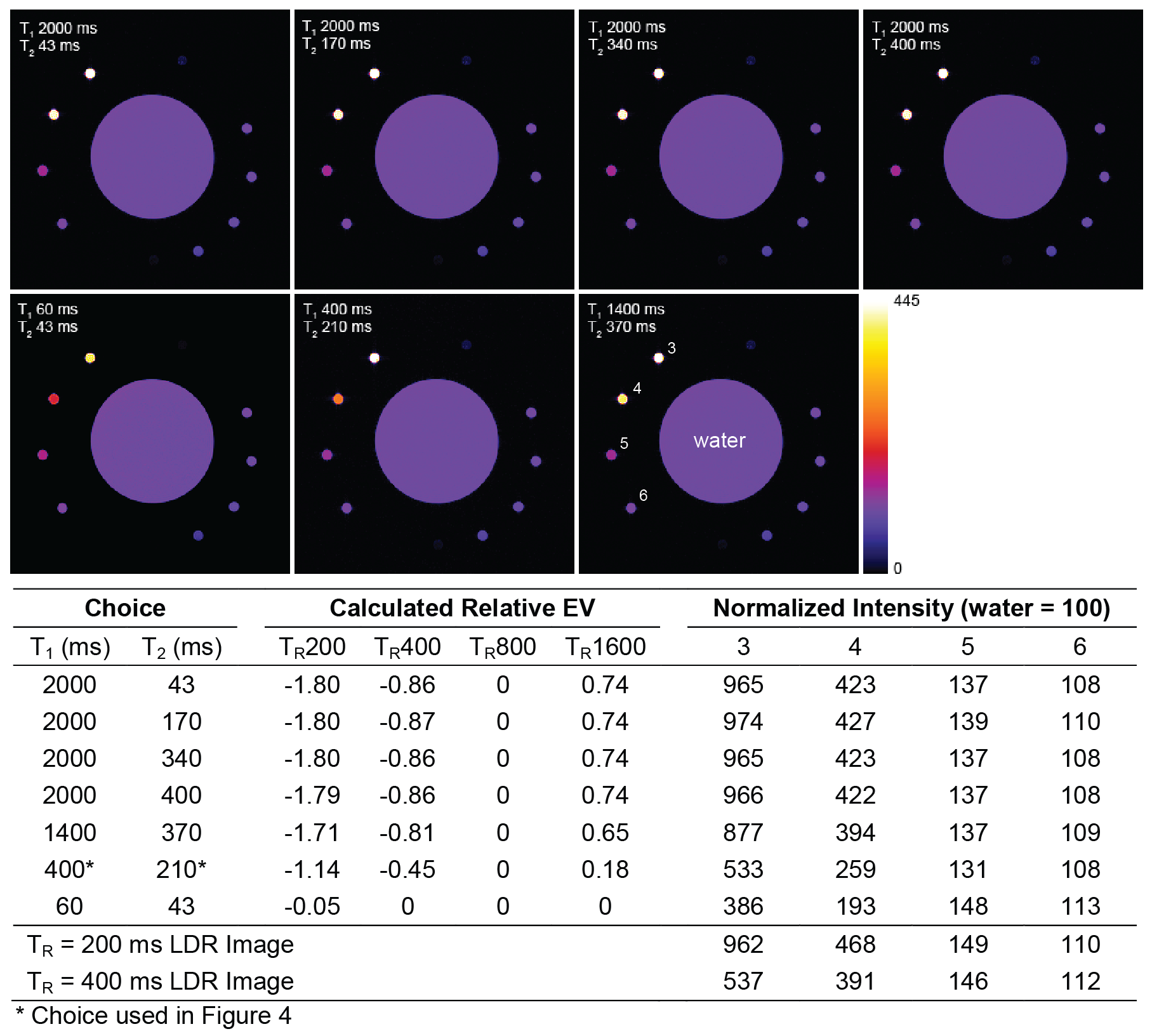

Supplement: Figure S3 — Effects of the choice of T1 and T2 in calculating EV on T1-weighted HDR processing. The values tested are the T1 and T2 of the samples imaged. The choice of T2 has almost no effect while the choice of T1 has moderate effect. In general, the quantitative contrast changes, but the relative order of the feature intensities is preserved regardless of the T1 chosen for EV calculation. The variations caused by the different choices of T1 are similar to variations seen in conventional T1-weighted imaging due to different TR settings. Water signal has been normalized to 100 across all images. (TIF) [file pone.0077883.s003.tif]

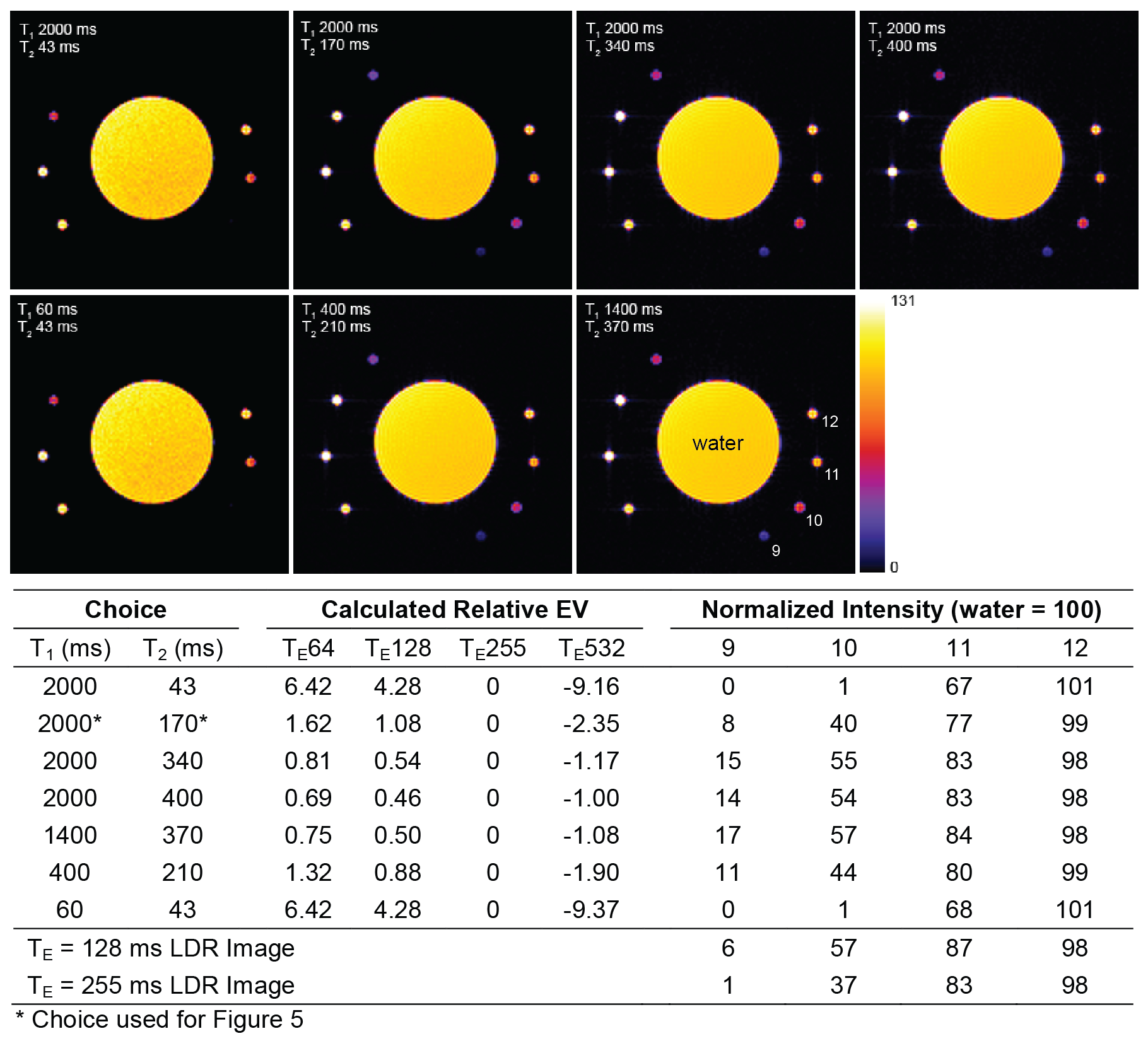

Supplement: Figure S4 — Effects of the choice of T1 and T2 in calculating EV on T2-weighted HDR processing. The values tested are the T1 and T2 of the samples imaged. The choice of T1 has almost no effect while the choice of T2 has moderate effect. In general, the conclusion is the same as for T1-weighted HDR processing. The quantitative contrast changes, but the relative order of the feature intensities is preserved regardless of the T2 chosen for EV calculation. The variations caused by the different choices of T2 are similar to variations seen in conventional T2-weighted imaging due to different TE settings. Water signal has been normalized to 100 across all images. (TIF) [file pone.0077883.s004.tif]

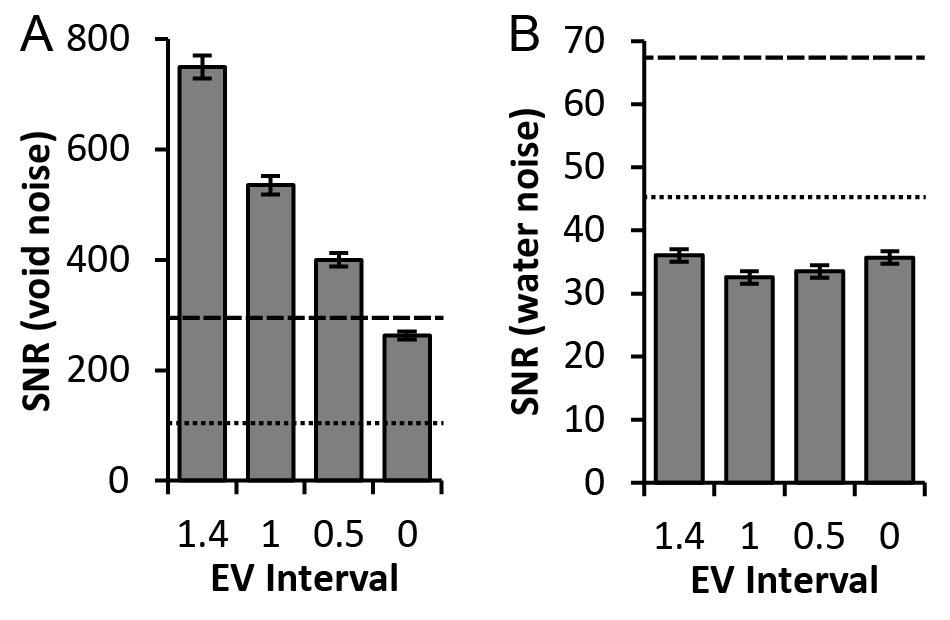

Supplement: Figure S5 — SNR comparison between image averaging and HDR-MRI. Data from Figure 6 was used for the analysis. Dashed line (----) and dotted line (····) represent the SNR of the averaged LDR images with TE 11 ms and TE 521 ms, respectively. (A) When calculated against void noise, HDR-MRI SNR improved with increasing EV interval and outperformed averaging. (B) When calculated against noise in the water signal, HDR-MRI SNR remained constant across EV intervals and underperformed averaging. SNR is inhomogeneous in HDR-MRI because the characteristic curve is nonlinear and each voxel is processed by the algorithm independently. N = 4 was used for the averaged images. Error bars represent standard deviation. (TIF) [file pone.0077883.s005.tif]
