# Supplementary material for: High Dynamic Range Processing for Magnetic Resonance Imaging
Source: PLoS One. 2013 Nov 8;8(11):e77883. doi: 10.1371/journal.pone.0077883 (PMC3826760; doi:10.1371/journal.pone.0077883)
Supplement: Table S1 — T1 and T2 used in the calculation of EV parameters for HDR processing. (DOCX) [file pone.0077883.s006.docx]

**Table S1.** T_1_ and T_2_ used in the calculation of EV parameters for HDR processing

| **Figure** | **T_1_ or T_2_ range (ms)** | **T_1_ or T_2_ chosen (ms)*** | **T_R_ or T_E_ series (ms)** | **Calculated EV^†^** |
| --- | --- | --- | --- | --- |
| 1 (T_1_) | T_1_ = 200 – 3000 | T_1_ = 1000 | T_R_ = 300, 1500, 2500 | 0, 1.58, 1.83 |
| 1 (T_2_) | T_2_ = 50 – 2000 | T_2_ = 750 | T_E_ = 200, 750, 1500 | 2.5, 1.45, 0 |
| 4 | T_1_ = 59 – 1922 | T_1_ = 400 | T_R_ = 200, 400, 800, 1600 | -1.14, -0.45, 0, 0.18 |
| 5 | T_2_ = 43 – 404 | T_2_ = 170 | T_E_ = 64, 128, 255, 532 | 1.62, 1.08, 0, -2.35 |
| 6 | T_2_ = 43 – 404 | T_2_ = 170 | T_E_ = 11, 181, 351, 521 | 0, 1.4, 2.8, 4.2 |
|  |  |  | T_E_ = 96, 213, 330, 447 | 0, 1, 2, 3 |
|  |  |  | T_E_ = 181, 245, 298, 362 | 0, 0.5, 1, 1.5 |
|  |  |  | T_E_ = 277, 277, 277, 277 | 0, 0, 0, 0 |
| 7 | T_2_ = ~ 10 – 70 | T_2_ = 50 | T_E_ = 21, 74, 138, 202 | 5.19, 3.66, 1.81, 0 |

***** T_1_ and T_2_ chosen represent intermediate values within the estimated range. Only the choice of T_1_ significantly affected the calculation of EV in T_1_-weighed imaging because T_E_ << T_2_ and was fixed across the T_R_ series (Equation 11 and Figure S3). Similar logic applies to T_2_-weighted imaging (Figure S4). See discussion section for further details.

**^†^** EVs (exposure values) were calculated using Equations 11 and 12 based on the T_1_ or T_2_ chosen for each figure. EV is relative and can be offset by any arbitrary constant without impacting HDR processing.
